# Modeling Side Chains in the Three-Dimensional Structure of Proteins for Post-Translational Modifications

**DOI:** 10.3390/ijms241713431

**Published:** 2023-08-30

**Authors:** Denis V. Petrovskiy, Kirill S. Nikolsky, Vladimir R. Rudnev, Liudmila I. Kulikova, Tatiana V. Butkova, Kristina A. Malsagova, Arthur T. Kopylov, Anna L. Kaysheva

**Affiliations:** Institute of Biomedical Chemistry, 119121 Moscow, Russia; petro2017@mail.ru (D.V.P.); kirill.s.nikolsky@yandex.ru (K.S.N.); v.r.rudnev@gmail.com (V.R.R.); likulikova@mail.ru (L.I.K.); t.butkova@gmail.com (T.V.B.); a.t.kopylov@gmail.com (A.T.K.); kaysheva1@gmail.com (A.L.K.)

**Keywords:** modeling side chains, non-canonical amino acid, post-translational modifications, rotamer library, phosphorylation

## Abstract

Amino acid substitutions and post-translational modifications (PTMs) play a crucial role in many cellular processes by directly affecting the structural and dynamic features of protein interaction. Despite their importance, the understanding of protein PTMs at the structural level is still largely incomplete. The Protein Data Bank contains a relatively small number of 3D structures having post-translational modifications. Although recent years have witnessed significant progress in three-dimensional modeling (3D) of proteins using neural networks, the problem related to predicting accurate PTMs in proteins has been largely ignored. Predicting accurate 3D PTM models in proteins is closely related to another fundamental problem: predicting the correct side-chain conformations of amino acid residues in proteins. An analysis of publications as well as the paid and free software packages for modeling three-dimensional structures showed that most of them focus on working with unmodified proteins and canonical amino acid residues; the number of articles and software packages placing emphasis on modeling three-dimensional PTM structures is an order of magnitude smaller. This paper focuses on modeling the side-chain conformations of proteins containing PTMs (nonstandard amino acid residues). We collected our own libraries comprising the most frequently observed PTMs from the PDB and implemented a number of algorithms for predicting the side-chain conformation at modification points and in the immediate environment of the protein. A comprehensive analysis of both the algorithms per se and compared to the common Rosetta and FoldX structure modeling packages was also carried out. The proposed algorithmic solutions are comparable in their characteristics to the well-known Rosetta and FoldX packages for the modeling of three-dimensional structures and have great potential for further development and optimization. The source code of algorithmic solutions has been deposited to and is available at the GitHub source.

## 1. Introduction

Amino acid substitutions and post-translational modifications (PTMs) are critical to the function of many proteins in living systems, and understanding their effects at the molecular level is important for both basic and applied research in biology and medicine [1,2]. Post-translational modifications of proteins, such as phosphorylation, acetylation, methylation, carboxylation, and hydroxylation, play a key role in cell ontogeny [3,4]. For example, PTMs play an important role in regulation of enzyme activity, protein transport, and changing of protein stability [5,6]. Non-enzymatic PTMs, such as carbonylation and oxidation, often occur as a consequence of oxidative stress and are considered a ubiquitous mechanism for non-specific protein damage associated with age-related disorders, including neurodegenerative diseases, cancer, and diabetes mellitus [7,8]. It is important to note that amino acids often undergo significant changes in their physicochemical properties upon modification, which sometimes dramatically alters the structure of the affected protein and its dynamics and ability to interact with the environment and other proteins [3,9].

One of the key challenges in modeling 3D protein structures for amino acid substitutions and post-translational modifications is predicting the correct conformations of amino acid side chains in proteins, also called “packing” [10]. Most of the currently available side-chain packing methods can be roughly divided into two large groups.

The first group is the protein physics-based approaches that involve searching within a given sample space, often defined by a library of predefined rotamers. A rotamer (short for “rotational isomer”) is a single side-chain conformation represented as a set of values, one for each degree of freedom of the dihedral angle. The side chains of proteins usually exist in a limited number of low-energy conformations, and these conformations are contained in rotamer libraries. Rotamer libraries typically contain information about the conformation, the frequency of a particular conformation, and the variance of dihedral mean values that can be used in searches or sampling. One of the most famous and frequently used libraries today is the Dunbrack library [11]. This group of methods looks at the problem from a physicochemical point of view and tries to optimize the interactions between side chains, avoiding steric collisions and minimizing the overall energy of the system.

The second group uses machine learning methods to reconstruct amino acid side chains. These methods use deep neural networks or neural network ensembles to model the position of side chains [12,13,14,15]. Some solutions use a combination of machine learning and rotamer library space search to determine the optimal side-chain conformation. A number of solutions use neural networks to find optimal side-chain scoring functions and use these functions to search for side-chain conformations in the rotamer library [16].

All methods for predicting side-chain conformations show good results for canonical amino acid residues, but for non-canonical amino acid residues (PTMs), there exists a practical problem hindering progress in this area. The problem is that the Protein Data Bank (PDB, https://www.rcsb.org/, accessed on 5 June 2023) contains significantly less data on PTM residues than on canonical amino acid residues. For comparison, while the number of residues of canonical amino acids is measured in millions, the number of residues modified by a particular type of PTM is in the best-case scenario measured in thousands of units and on average hundreds or even tens. This amount is not enough for training neural network models or building rotameter libraries with full-fledged statistical potential. This explains the relatively small number of solutions for the incorporation and packaging of post-translational modifications into the 3D protein structure. Rosetta and FoldX are the most famous and widespread packages currently providing PTM modeling and repacking.

In this study, we consider a number of algorithms for choosing the optimal position of side chains from an ensemble of rotamers for protein structures with PTMs. The algorithms are evaluated for a large test set of proteins, and their performance is compared with that of the well-known Rosetta and FoldX protein structure modeling packages. We also discuss the advantages and drawbacks of the algorithms and point out possible improvements and extensions to our methods.

## 2. Results

We carried out a comprehensive analysis aimed to evaluate the performance of algorithms purposed for the modeling and reconstructing of PTMs and canonical amino acid residues in three-dimensional protein structures:

- Monte Carlo Markov Chain (MCMC) sampling (rotamer) using rotamer libraries. Dunbrack rotamer libraries were used for canonical amino acid residues, and proprietary libraries were assembled for five common post-translational modifications.
- Monte Carlo Markov Chain (MCMC) sampling (off-rotamer): This algorithm allows side-chain torsion angles to go beyond the values of the rotamer library. The rotamer library is used only to control the degree of changes in angles.
- Generative algorithm (GA-rotamer) is an evolutionary search algorithm with initialization of the initial population from the rotamer library.
- Generative algorithm (GA-random) is an algorithm with initialization of the initial population from a uniform distribution. The rotamer library is not used in this algorithm.

A detailed description of these algorithms is available in Section 4.

We also compared outcomes obtained by these algorithms and the well-known modeling services Rosetta and FoldX. Since our work is more focused on the prediction of side-chain conformations caused specifically by PTMs and their neighborhoods, to achieve satisfactory quality, we took a set of high-resolution (≤1.5 Å) PDB structures (total 100 structures) carrying each type of considered PTM function (complete list of advised set of structures is available in Supplementary Table S3).

The evaluation algorithm was built as follows:

- All side chains were removed from the PDB structure.
- All side chains were restored, and side chains were repackaged within a radius of 10 Å from the mutation point using the algorithms described before.
- For the restored structure, the quality indicators provided by the MolProbity service [1] (Table 1), RMSD indicators, and torsion angle were calculated for the comparison with the original structure.

A similar algorithm was used to assess performance with the Rosetta and FoldX packages: the side chains were recovered and metrics were calculated for the recovered structures. Since the FoldX software package does not support the PTM part, the corresponding positions in the tables and plots are not filled.

The MolProbity service was chosen to control integrity characteristics of the restored structures and provides metrics for the assessing of the quality of structures (Table 1). Hydrogen atoms were added to and possible inversions of the side chains of asparagine, glutamine, and histidine were recognized and accepted.

**Figure 1:**
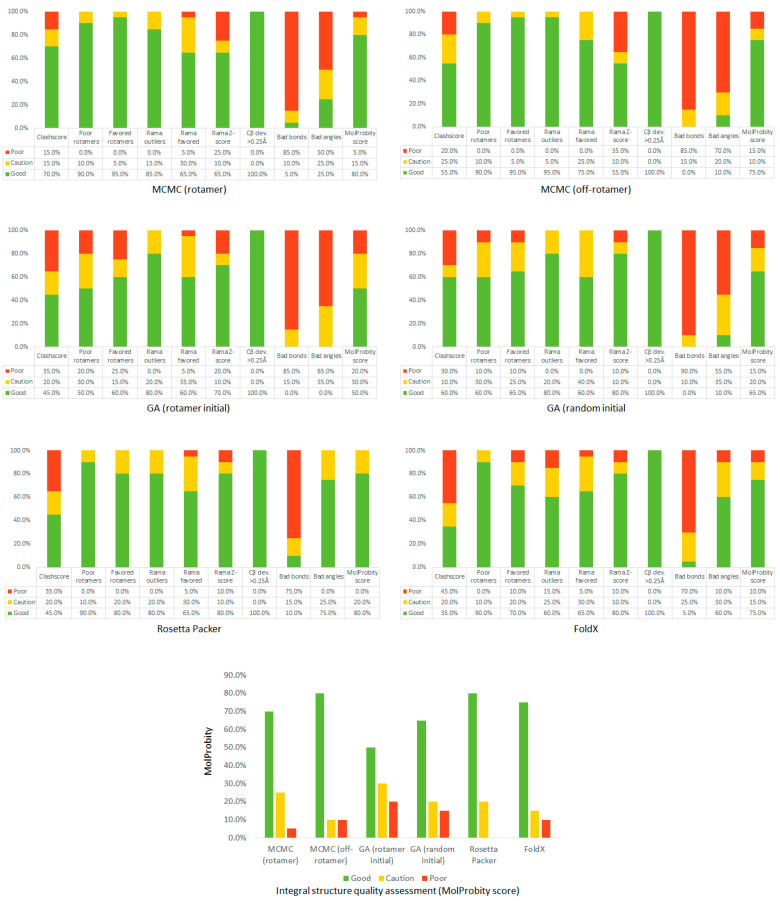
Comparison of structure quality indicators provided by the MolProbity service for MCMC (rotamer), MCMC (off-rotamer), GA (rotamer initial), GA (random initial), Rosetta Packer, and FoldX. Indicators are calculated for 100 PDB with high-resolution (≤1.5 Å) structures of the validation set.

Result comparisons between the in-house algorithms and Rosetta or FoldX were consequently handled using the MolProbity service to elucidate the quality of calculated structures (Figure 1).

We also determined typical deviations in the structures of amino acid residues for each algorithm and established those residues where deflection incidents were the most frequent.

We defined such residuals with deviations as “marginal” if such residuals matched one of the following provisions:

- Abnormally closely located atoms;
- Going beyond the allowable values of the Ramachandra map;
- Abnormal angles or out of angles of the rotamers.

**Figure 2:**
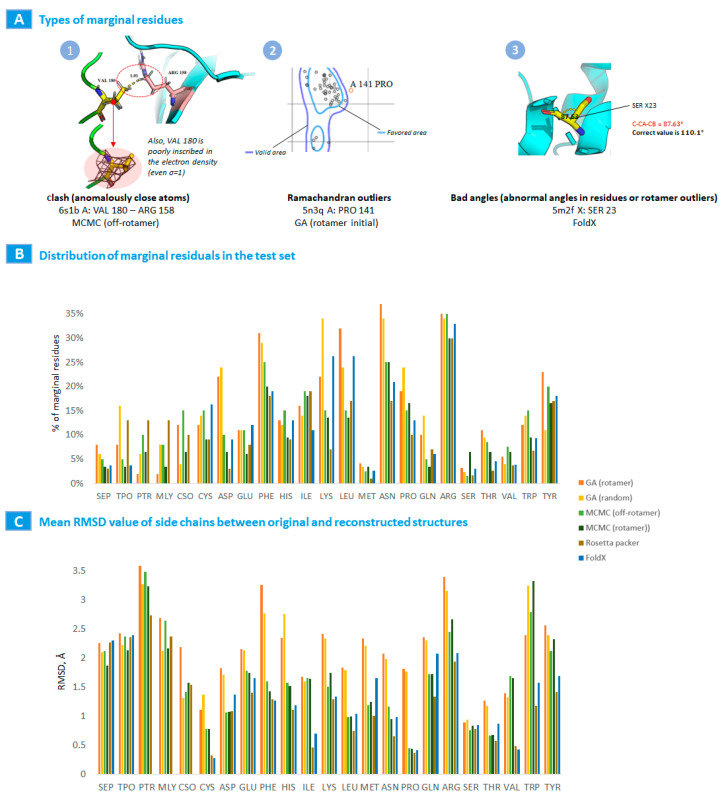
Comparison of algorithm results for MCMC (rotamer), MCMC (off-rotamer), GA (rotamer initialization), GA (random initialization), Rosetta Packer, and FoldX. (**A**) Types of the most common marginal amino acid residues with examples from the test set (PDB ID: 6s1b A-180 VAL, 5n3q A-141 PRO, 5m2f X-23 SER). (**B**) Distribution of marginal amino acid residues by test set. (**C**) Distribution of mean RMSD values between original and reconstructed structures.

The defined marginal amino acid residues among plenty of structures in the test data set were extracted, and deviations classified by the PTM type and canonical amino acids for each algorithm were estimated and ranged (Figure 2) with an average calculated RMSD (Table S1 in the Supplementary Materials).

It is also interesting to look at the comparison of mean absolute errors (MAEs) of torsion angles χ in different methods (Table S2 in the Supplementary Materials).

Gathering the obtained data, we found that the tested in-house algorithms demonstrated well results that are not at odds with the well-known Rosetta and FoldX packages, and some were better for PTMs. Some outliers in MolProbity indicators could be observed for PHE, ASN, and ARG residues. However, these emissions are typical of all considered algorithms and software packages, which may indicate that the reference model in MolProbity imposes excessive quality requirements.

**Figure 3:**
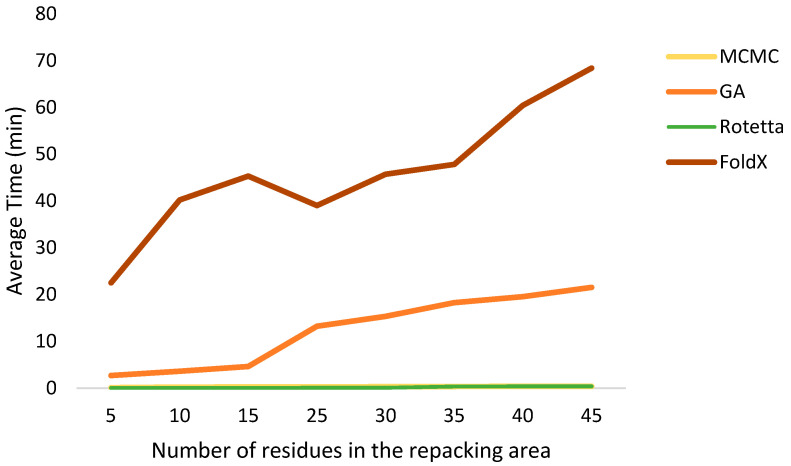
Average running time of algorithms, depending on the number of residues in the repacking area (GA population size = 300, number of generations = 40). CPU AMD Ryzen 5 4000. For MCMC algorithms, this plot reflects MCMC (rotamer) and GA (random) for GA since the speeds within the group are approximately the same.

If we compare the speeds of these algorithms, it should be immediately noted that the FoldX software package takes more computing time than all other algorithms. A comparison of the operation speed is presented in Figure 3.

The following conclusions can be drawn from the presented comparative data.

1. The best results in our study, in terms of both accuracy and processing speed, were demonstrated by the Rosetta software package. This was expected, since Rosetta is one of the leading molecular modeling packages and is widely used by researchers around the world. According to the published documentation [17], Rosetta also uses the MCMC algorithm inside its software implementation, and the difference in performance apparently depends only on the selected scoring function.
2. The FoldX software package also generally shows good results, but its speed is much slower than that of all the algorithms considered. In addition, FoldX only supports two PTMs (SEP and TPO), and we could not fully evaluate its results.
3. The MCMC algorithm with sampling from the rotamer library shows good results, close to those of Rosetta, and even better for some PTMs.
4. The results of the MCMC off-rotamer algorithm are slightly worse but still acceptable. If we thoroughly analyze the results provided by this algorithm, we can observe that in some cases its performance is better than that of other algorithms, but no regular pattern could be identified.
5. The results of the work of genetic algorithms, despite the fact that their performance in general turned out to be worse than that of all the others, surprised us. The interesting point here is that GA initialized with random numbers from a uniform distribution works better than GA initialized from the rotamer library. This makes it possible not to use rotamer libraries at all for identifying the optimal position of side chains and obtain results with quite acceptable accuracy, which is especially important for rare non-canonical amino acid residues. If we analyze in detail the results of the work of GA algorithms, we can observe a picture similar to that for the MCMC off-rotamer: some structures are determined better compared to other algorithms, while some are worse. In general, the results of GA work are unstable, but as it seems to us these algorithms show great promise for solving this problem.

We assume that genetic algorithms have a great potency to cover modeling of three-dimensional protein structures, although they are still rarely applied in this realm. We also noticed that as the resolution of protein structures increases, the accuracy of all algorithms, including Rosetta Packer and FoldX, drops dramatically (Figure S1 in the Supplementary Materials), while the accuracy of GA severely increases. This can be caused by the fact that the electron density in structures with a low resolution and poor quality is closer to the posterior distribution of the rotamer libraries used for sampling. The genetic algorithm initialized from uniform distribution does not use rotamer libraries, and for structures with good resolution, its predictions are closer to the experimental data.

Currently, we are working under the following main hurdles:

1. Improving the overall accuracy of the genetic algorithm. According to our preliminary studies, perfect improvement of accuracy can be achieved using the particle swap optimization (PSO) [3] approach, where the elements of the search space (in our case, atoms of amino acid residues) interact without centralized coordination.

2. Reproducibility of results. Since genetic algorithms are inherently heuristic, the stability of their results is not guaranteed. To ensure stable reproducibility, we are working toward the integration of GAs and neural networks, where neural networks implement the functions of genetic operators and evaluation functions.

## 3. Discussion

We developed a solution for building a library of rotamers for PTMs and any non-canonical amino acid residues present in the Protein Data Bank. We also implemented and conducted a comparative analysis of the algorithms for side-chain reconstruction and “repacking”:

1. Monte Carlo Markov Chain (MCMC) sampling (rotamer) using rotamer libraries. Dunbrack rotamer libraries were used for canonical amino acid residues, and proprietary libraries were assembled for five common post-translational modifications.

2. Monte Carlo Markov Chain (MCMC) sampling (off-rotamer): This algorithm allows side-chain torsion angles to go beyond the values of the rotamer library. The rotamer library is used only to control the degree of changes in angles.

3. Generative algorithm (GA-rotamer) is an evolutionary search algorithm with initialization of the initial population from the rotamer library.

4. Generative algorithm (GA-random) is an algorithm with initialization of the initial population from a uniform distribution. The rotamer library is not used in this algorithm.

The conducted comparative analysis shows that the most accurate results are obtained by uses the MCMC algorithm using rotamer libraries (MCMC-rotamer). This was not surprising, since this algorithm is classic for solving problems of this kind and is used everywhere, including in such well-known software packages as Rosetta. The MCMC off-rotamer algorithm yields results comparable with those of the MCMC-rotamer algorithm; in general, it cannot be said that this methodology affects accuracy. For PTM amino acid residues, accuracy scores were either equal to or better than those of Rosetta, indicating that the rotamer libraries have been assembled with high quality.

**Figure 4:**
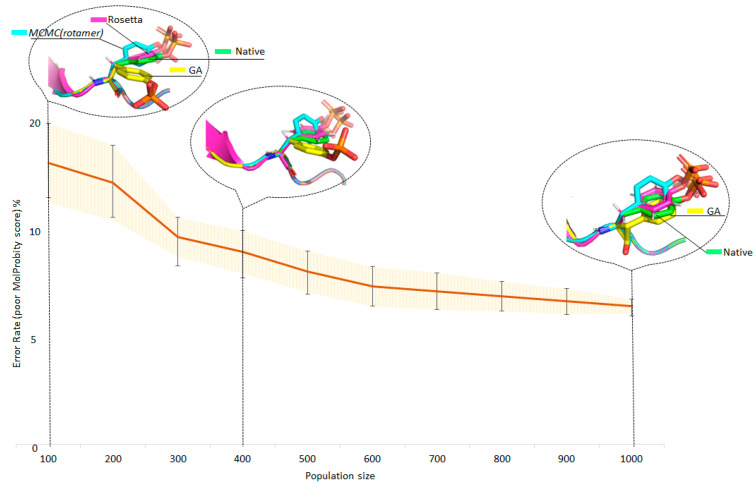
Improving the accuracy of the genetic algorithm by increasing the population size (GA-random): 20 PDB structures containing O-phosphotyrosine, population change step = 100, repetitions at each step = 10. Example: PDB ID: 2qon (A-701 O-phosphotyrosine).

Among GA algorithms, one should separately single out the algorithm with initialization of the initial population from a uniform random distribution, since this methodology allows one to completely give up using rotamer libraries. Although the performance of genetic algorithms is generally worse than that of the MCMC family, we deem that these algorithms have great potential for further research and development. Thus, one obvious way to improve the accuracy of GA side-chain modeling is to increase the population size. In Figure 4, we plotted the relationship between population size and error rate (poor MolProbity score) for PTM O-phosphotyrosine. The experiment involved 20 PDB structures containing O-phosphotyrosine; the population increased by 100 individuals, and 10 technical repetitions were performed at each step.

As one can see in the diagram shown in Figure 4, the accuracy of GA increases with population size. As the population size rises, the speed of the algorithm decreases simultaneously, mainly due to the multiple increase in computational costs in the function of assessing the fitness of each individual. This problem is solved well by parallelizing the evaluation task on several CPUs. The ability to parallelize computations is one of the important advantages of genetic algorithms.

Among the shortcomings of genetic algorithms, there is relative instability of the search for solutions: they may differ each time the algorithms are run. This problem is inherent in all heuristic search and optimization algorithms and can potentially be solved by integrating genetic algorithms and neural networks (neuro-genetic networks).

We will continue our research in this direction and will present new research in this area in future papers.

## 4. Materials and Methods

### 4.1. Rotamer Library

Our solution implements the functionality of building a rotamer library for any amino acid residue present in the PDB (https://www.rcsb.org/). To build a library, one needs to indicate the code of the amino acid residue and the possible torsion angles that groups of atoms can form. The code of the residue is searched across the PDB, and a library of rotamers is formed, consisting of a set of low-energy conformers and their associated internal energies generated using CREST [18]. For canonical amino acid residues, the Dunbrack library [19] was used, and for the most common PTMs, our own rotamer libraries were collected from the PDB (available at https://figshare.com/s/253d32313e1294fbf1e2, accessed on 5 June 2023).

Multiple entries in the same PDB file were treated as different entries. Our solution allows one to build a library for any PTM of non-canonical amino acid residues. For the PTM presented in Table 2, testing and debugging of algorithms for finding the optimal conformation of side chains of both the modifications per se and its neighbors (repacking) were carried out.

### 4.2. Side-Chain Modeling and Repacking

In single-site mutants and closely related proteins, the backbone usually changes little, and a prediction of the target structure can be made by accurately predicting the position of side chains. When modeling mutations, it is important to model not only changes at the mutation point per se but also changes in conformations of neighboring side chains (perform local “repacking” of neighboring side chains). In this article, we describe and compare three modeling and repacking algorithms.

### 4.3. Markov Chain Monte Carlo (MCMC) Sampling from the Rotamer Library

This classic method uses Markov Chain Monte Carlo (MCMC) sampling to repackage all amino acid residues within a user-specified radius using a rotamer library. The algorithm is the most common variant for solving problems of this kind; it has been described quite well in [20] and used in many libraries and software products, such as Rosetta. Markov Chain Monte Carlo sampling can be described as follows:

1. The user defines the number of selection steps and the neighborhood radius from the mutation point (by default, R = 10.0 A).

2. At each sampling step, a site is randomly selected from a user-defined radius. For a given site, dihedral angles of the side chain of the site and the average deviation of this angle are randomly selected from the rotamer library.

3. The step is accepted or rejected using the Metropolis–Hastings criterion [21] based on the energy function. The clash evaluation function based on flat-top Lennard–Jones potential energy is used as an evaluation function in our algorithms. The interaction energy in this function consists of repulsive and attractive van der Waals terms and is defined as:

(1) $$E\_vdw\;\left(d\right)=\{\begin{array}{c} \begin{array}{c} 10,\;\frac{d}{\sigma_{ij}}\;\leq \;0.8254\;\\ \begin{array}{c} 57.273\left(1-\frac{d}{\sigma_{ij}}\right),\;0.8254\;\leq \;\frac{d}{\sigma_{ij}}\leq 1\;\\ E_{ij}{\left(10-9\frac{d}{\sigma_{ij}}\right)}^{\frac{57.273}{9\;E_{ij}}}-E_{ij},\;1\leq \;\frac{d}{\sigma_{ij}}\leq \;\frac{10}{9}\; \end{array} \end{array}\\ \frac{E_{ij}}{4}{\left(9\frac{d}{\sigma_{ij}}-10\right)}^{2}-E_{ij},\;\frac{10}{9}\leq \;\frac{d}{\sigma_{ij}}\leq \;\frac{4}{3}\;\\ 0,\;\frac{d}{\sigma_{ij}}\;\geq \;\frac{4}{3}\; \end{array}\;$$

where $E_{i}$ are the values from the CHARMM param19 potential [22] and *d* is the distance between the two atoms. This scoring function is used in the popular SCWRL4 side-chain conformation modeling software package [23].

### 4.4. Markov Chain Monte Carlo Sampling outside the Rotamer Library

This method implements an algorithm for selecting side-chain conformations with deviations from canonical dihedral angles from fixed rotamer libraries. The sampling algorithm is described as follows:

1. The user defines the number of sampling steps and the radius (by default, R = 10.0 A).
2. At each sampling step, a site is randomly selected from a user-defined radius. For a given site, dihedral angles of the side chain of the site and the average deviation of this angle are randomly selected from the rotamer library.

The new dihedral angle values of side chains are defined using a random sample from the von Mises distribution [24], with the center equal to the dihedral angle in the rotamer library and dispersion reciprocally proportional to squared deviation. This can be formally described as follows:

(2) $$p\left(x\right)=\frac{e^{k\cos\left(x-\mu \right)}}{2\pi I_{0}\left(k\right)}$$

where *μ* is the mode and *k* the dispersion (*k* = 1/*σ*^2^, *σ*^2^—std from the rotamer library), and *I*_0_ is the modified Bessel function of order 0. The von Mises distribution (also known as the circular normal distribution) is a continuous probability distribution on a circle. By applying additional sampling from the von Mises distribution, we can expand the search space for rotamers, which is especially true for rotamers with low statistical potential, such as PTMs. Like in the first algorithm, the step is accepted or rejected using the Metropolis–Hastings criterion [21] based on the energy function.

### 4.5. Modeling Using a Genetic Algorithm

Genetic algorithms are a family of search algorithms whose ideas are based on the principles of natural evolution. Genetic algorithms implement a simplified version of Darwinian evolution:

- Variability—the characteristics of individual individuals that are part of the population may change;
- Heredity—some traits are consistently transmitted from an individual to their descendants;
- Natural selection—better-adapted individuals are more successful in struggling for survival and leave more offspring in the next generation.

In our work, we considered a variant of solving the problem of finding the optimal side-chain conformation (“repacking”) for PTM and amino acid substitution and its neighboring regions within a user-specified radius using a genetic algorithm. We decided to analyze the possibility of solving the problem using a genetic algorithm for two reasons:

1. Genetic algorithms are rarely used to solve this problem. According to our hypothesis, they can show good results, especially for amino acid residues with a small statistical potential of rotamer libraries due to the greater variability of solutions formed during mutations and crossing.
2. Genetic algorithms have a number of advantages over traditional search and optimization algorithms:
  - Ability to perform global optimization;
  - Applicability to problems with complex mathematical representation;
  - Resistance to noise;
  - Support for parallelization and distributed processing.

The proposed genetic algorithm for solving the problem of finding the optimal conformation is described in the following sections.

#### 4.5.1. Creating the Initial Population

The initial population is a set of individuals, each being represented by a set of chromosomes (a sequence of dihedral angles). The dihedral angles to create the population are either randomly selected from a library of rotamers or selected from a uniform distribution of the range (−π, π). The method of specifying the initial population is determined by the user. When evaluating the algorithm efficiency, we consider both options for the formation of the initial population.

#### 4.5.2. Selection

Individuals are selected from the current population in such a way that preference is given to the best ones. This is performed at the beginning of each cycle operation, and individuals are selected from a population that will become parents for the next generation. Selection is probabilistic in nature, and the probability of choosing an individual depends on their fitness. In our solution, a selection method called “tournament” is used:

1. *k* Randomly selected individuals from the population participate in each round of selection.
2. The individual whose fitness is higher wins and is selected to form the next generation.
3. The process is repeated until the number of “parents” becomes equal to the population size.

The number of individuals participating in each round of the tournament (parameter *k*) is called the tournament size. The larger the tournament size, the higher the chances that the best representatives of the generation will participate in the rounds, and the less likely that individuals with low fitness will win the tournament and qualify for the next generation. In our solution, we set the tournament size at 1/20 of the population size.

Furthermore, we use the elitism strategy when selecting and forming the population. The elitism strategy allows one to transfer a certain percentage of the best individuals to the next generation. Thus, it guarantees to a certain extent that the best individuals will not disappear from the solution due to mutations and crossbreeding. In our solution, we carry over the top 15% individuals to the next generation.

#### 4.5.3. Fitness Function

The clash evaluation function based on the flat-top Lennard–Jones potential energy (Equation (1)) was also used as the fitness function of an individual in a population.

#### 4.5.4. Crossing and Mutation

In the classic genetic algorithms, chromosomes are usually described by binary or integer representations and crossing and mutation operators are defined over sets of integers or binary numbers. In our algorithm, chromosomes represent dihedral angles and are described by real numbers. Therefore, in our algorithm, we use special crossing and mutation methods adapted to work with real numbers. It is also important to note that since the chromosomes are torsion angles in our case, we must ensure that the values of the angles lie within the region (−π, π).

##### Crossover Operators

The crossing or recombination operator corresponds to biological crossing during sexual reproduction. It is used to combine the genetic information of two individuals acting as parents in the production of two offspring. Crossing in our algorithm is applied with a probability of 0.9. In our algorithm, we use two crossing operators: mixing crossing and imitation binary crossing; these operators are chosen equiprobably.

1. Blend crossover (BLX): In the case of using this operator, each child is randomly selected from the interval formed by parents *parent*_1_ and *parent*_2_:

[*parent*_1_ − *α* (*parent*_2_ − *parent*_1_), *parent*_2_ + *α* (*parent*_2_ − *parent*_1_)]

where *α* belongs to the interval [0, 1] and determines the interval width. In our implementation, *α* = 0.5 is assumed, which is equivalent to doubling the interval.

2. Simulated binary crossover (SBX): The main idea behind this method is to simulate the properties of a single-point crossing, often used for binary representation of chromosomes, one of its properties being that the average value of the parents is equal to the average value of the offspring. In the case of SBX, two children are created from parents in the following way:

(a) *offspring*_1_ = 1/2 [(1 + *β*) *parent*_1_ + (1 − *β*) *parent*_2_];   (b) *offspring*_2_ = 1/2 [(1 − *β*) *parent*_1_ + (1 + *β*) *parent*_2_],

where *β* is a random number called the distribution coefficient.

This scheme has the following properties:

- The average of descendants is equal to the average of parents.
- When *β* = 1, the descendants are exact copies of the parents.
- When *β* < 1, the offspring are located closer to each other than the parents.
- When *β* > 1, the offspring are further apart than the parents.

In order to preserve the similarity between descendants and parents, the parameter *β* must be chosen and distributed with a high probability density in the vicinity of 1. In our implementation, the value of the parameter *β* is calculated using another random variable µ, which is uniformly distributed in the interval [0, 1]:

$$\beta ={\left(2\mu \right)}^{\frac{1}{\eta +1\;}},\;\mu \leq \;0.5$$

(3) $$\beta ={\left(\frac{1}{2}\left(1-\mu \right)\right)}^{\frac{1}{\eta +1\;}},\;\mu >0.5$$

The parameter *η* is a constant called the distribution index or the crowding factor. The larger the value of this parameter, the more similar the descendants to their parents. In our implementation, the value of this parameter is set to *η* = 12 by default and can be configured by the user.

The control over the boundaries of values by chromosomes in the solution is implemented as follows: if the value of the descendant lies outside the boundaries of the interval (−π, π), then its value is set equal to the nearest boundary of the interval (−π or π, respectively).

##### Mutation Operators

The mutation in our decision scheme is the last genetic operator applied to create a new generation. It applies to the offspring produced as a result of selection and crossing. The mutation operation is probabilistic and is typically used quite rarely, since it can degrade the quality of an individual and lead to degeneration of the genetic algorithm into a random search. In our algorithm, the default mutation rate is set to 0.15 and is user-configurable. As a mutation operator in genetic algorithms with real coding, a sample is used from a distribution, ensuring that the offspring is in relative proximity to the parents.

Our solution implements two types of mutations applied with equal probability:

1. Mutation by a sample from the von Mises distribution, with a center equal to the value of the angle in the chromosome and a variance inversely proportional to squared deviation (*σ*). The squared deviation is either selected from the rotamer library if the initial population was formed from the rotamer library, or the value is randomly selected from the uniform distribution (0, k), and then crossing and mutation operations are also performed for the value of *σ*.

2. Mutation using an operator in which the distribution density is given by a polynomial function [25]. The range of values of the polynomial density function is confined to the interval (−π, π).

**Figure 5:**
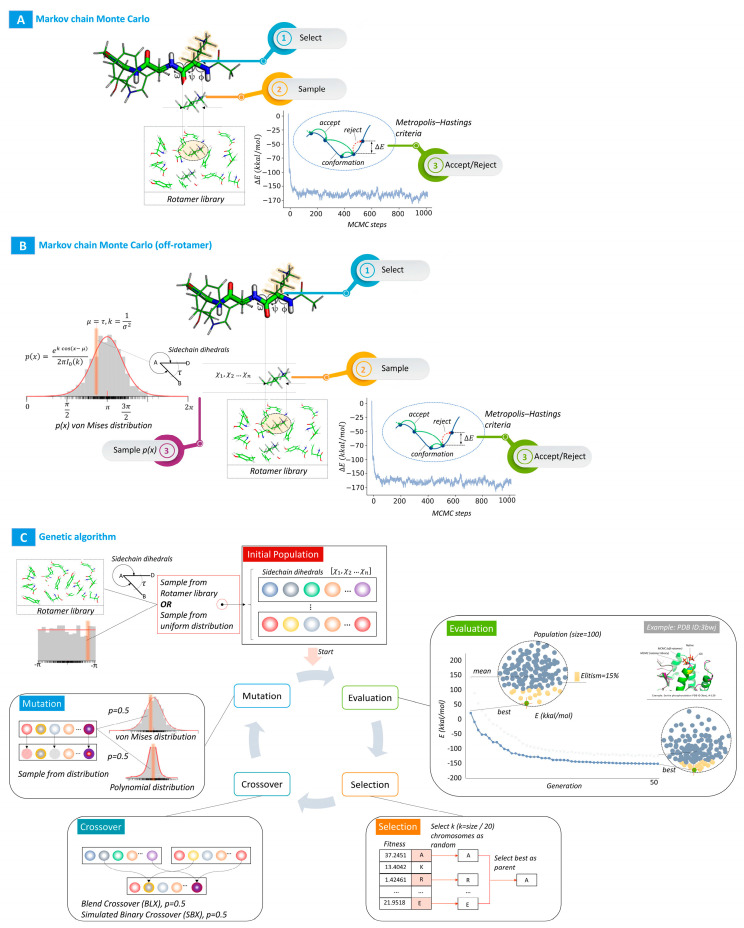
(**A**). Markov Chain Monte Carlo (MCMC) sampling from a rotamer library. (**B**). Off-rotamer MCMC sampling. (**C**). The process of genetic algorithms. The generation convergence plot shows a real example corresponding to PDB ID 3bwj (A-139 SER phosphorylation).

The generalized scheme of the described algorithms is shown in Figure 5.

## 5. Conclusions

Amino acid substitutions and post-translational modifications (PTMs) are essential to the function of many proteins in organisms. One of the challenges in modeling 3D protein structures for amino acid substitutions and PTMs is predicting the correct conformations of amino acid side chains in proteins. In order to help research in this area, we developed a modular modeling library that allows one to build one’s own libraries of rotamers for standard and non-standard amino acid residues, as well as model side-chain conformations using various methods.

The library is open and available to a wide range of researchers for use, development, and elaboration of hypotheses. The library is used by researchers to predict side-chain conformations in projects or as a good starting point for molecular or quantum mechanical modeling of side-chain atoms for both standard and non-standard amino acid residues.

## Figures and Tables

**Table 1:** Structure quality indicators obtained using the MolProbity service.

Metric	Description	Reference		
		Good	Caution	Poor
Clashscore	Clashscore is the number of serious steric overlaps (>0.4 Å) per 1000 atoms. P—percentile.	P ≥ 66	66 > P ≥ 33	P < 33
Poor rotamers	Residues with side chains marginal in deviation from rotamers. Out—outlier %.	Out ≤ 0.3%	0.3% < Out ≤ 1.5%	Out > 1.5%
Favored rotamers	The percentage of amino acid residues that are in the preferred regions of the rotamers. Fav—favored % of the total.	Fav ≥ 98%	Fav ≥ 95%	Fav <95%
Ramachandran outliers	Ramachandran outliers—complete marginals on the Ramachandran map, the remains lie outside the allowed area of the map. Out—outlier % of the total.	Out ≤ 0.05%	0.05% < Out ≤ 0.5% Or Out 0.5% and Outlier count = 1	Out > 1.5% Or Outlier count ≥ 2
Ramachandran favored	The percentage of remnants that are in the preferred areas of the Ramachandran map. Fav—favored % of the total.	Fav ≥ 98%	Fav ≥ 95%	Fav < 95%
Ramachandran Z-score	Ramachandran Z-score validation checks the total Ramachandran distribution against the expected distribution [2].	abs(Z-score) ≤ 2%	2% < abs(Z-score) ≤ 3%	abs(Z-score) > 3%
Cβ deviations > 0.25 Å	Number of Cβ atoms with an unacceptable deviation from the expected position.	Outlier count = 0	0 < Outliers < 5%	Outliers ≥ 5%
Bad bonds	Number of covalent bonds that deviate significantly from the expected value. Out—outlier bond % of the total.	Out < 0.01%	0.01% ≤ Out < 0.2%	Out ≥ 0.2%
Bad angles	Number of bond angles that deviate significantly from the expected value. Out—outlier angle % of the total.	Out < 0.1%	0.1% ≤ Out < 0.5%	Out ≥ 0.5%
MolProbity score	Integral assessment of the quality of the structure according to the MolProbity service. The MolProbity score combines the clashscore, rotamers, and Ramachandran evaluations into a single score, normalized to be on the same scale as X-ray resolution. P—percentile.	P ≥ 66	66 > P ≥ 33	P < 33

**Table 2:** Frequent post-translational modifications (PTMs).

Precursor		PTM			
AA	Structure	PTM Code	Name	Structure	Total PDB Entry
SER	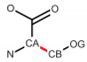	SEP	Phosphoserine	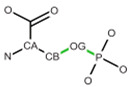	2437
THR	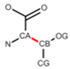	TPO	Phosphothreonine	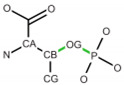	1864
TYR	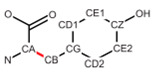	PTR	O-phosphotyrosine	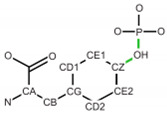	1423
LYS	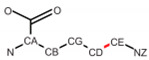	MLY	N-dimethyl-lysine	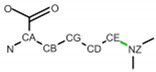	5296
CYS	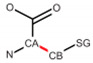	CSO	S-hydroxycysteine	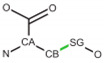	1552

## Data Availability

This article contains supporting information. Test data are available at https://figshare.com/s/253d32313e1294fbf1e2 (DOI: 10.6084/m9.figshare.23716644) (accessed on 5 June 2023). The source code is a free and open source Python API and is available at https://github.com/protdb/SCPacker.git (accessed on 20 July 2023).

## Supplementary Materials

The following supporting information can be downloaded at https://www.mdpi.com/article/10.3390/ijms241713431/s1.
