# Supplementary material for: Modeling Side Chains in the Three-Dimensional Structure of Proteins for Post-Translational Modifications
Source: Int J Mol Sci. 2023 Aug 30;24(17):13431. doi: 10.3390/ijms241713431 (PMC10488155; doi:10.3390/ijms241713431)
Supplement: Supplementary file 1 [file ijms-24-13431-s001.zip › ijms-2575760-SI.pdf]

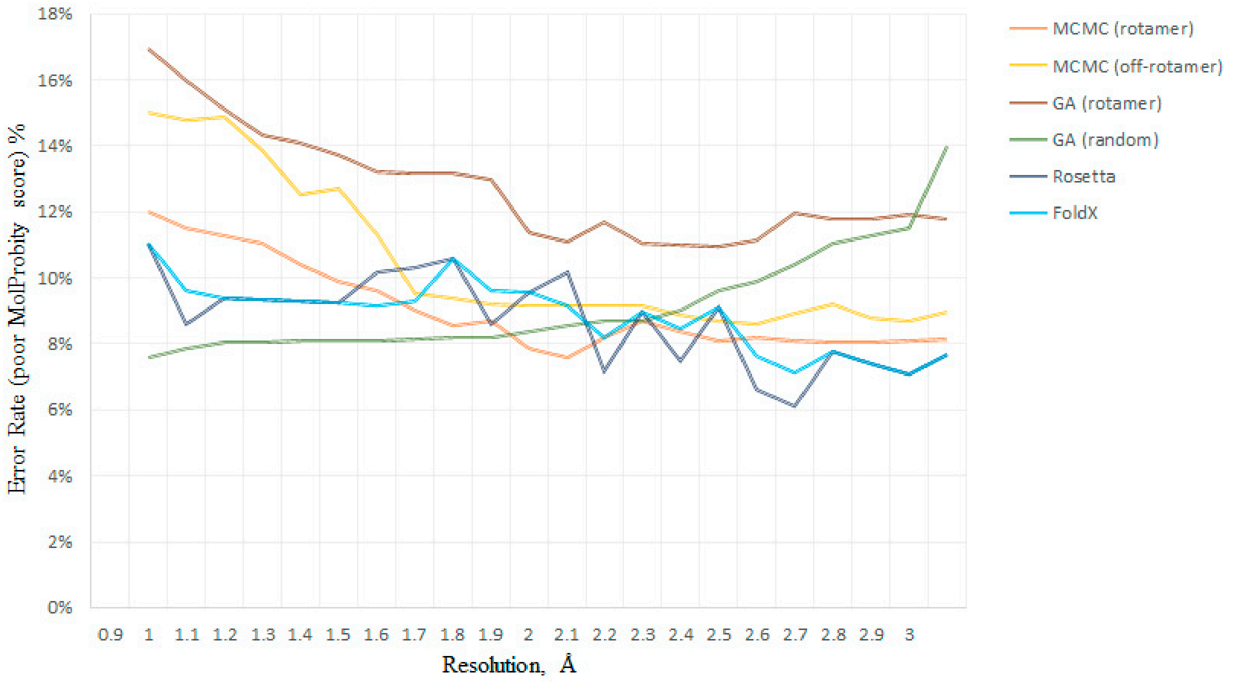

Figure S1. Dependence of Error Rate (poor MolProbity score, %) on structure resolution, Å. Graphs is based on 250 structures with different resolutions.

Table S1. Comparison between validation sidechain RMSD values for MCMC (rotamer), MCMC (off-rotamer), GA (rotamer initial), GA (random initial), Rosetta Packer and FoldX. The values for each amino acid are obtained by averaging RMSD values for 100 high-resolution (≤1.5 Å) PDB structures.

AA	Number of residues	RMSD, Å					
		MCMC (rotamer)	MCMC (off-rotamer)	GA (rotamer)	GA (random)	Rosetta packer	FoldX
SEP	20	1.87	2.12	2.26	2.09	2.27	2.3
TPO	20	2.13	2.37	2.42	2.22	2.36	2.39
PTR	20	3.23	3.48	3.59	3.27	**2.73**	-
MLY	20	2.16	2.64	2.69	2.12	2.37	-
CSO	20	1.57	1.41	2.19	1.31	1.54	-
CYS	474	0.77	0.77	1.11	1.37	0.32	0.27
ASP	299	1.07	1.06	1.82	1.71	1.08	1.37
GLU	414	1.74	1.78	2.15	2.13	1.4	1.65
PHE	193	1.42	1.6	3.26	2.77	1.29	1.27
HIS	231	1.51	1.57	2.35	2.76	1.1	1.18
ILE	354	1.64	1.65	1.68	1.6	0.46	0.7
LYS	312	1.74	1.5	2.41	2.33	1.29	1.33
LEU	359	0.99	0.98	1.83	1.79	0.74	1.04
MET	96	1.24	1.19	2.34	2.21	1.01	1.65
ASN	178	0.95	1.16	2.07	1.98	0.65	0.98
PRO	257	0.43	0.45	1.81	1.77	0.37	0.41
GLN	293	1.72	1.72	2.36	2.3	1.33	2.07
ARG	297	2.67	2.45	3.39	3.15	1.94	2.08
SER	392	0.83	0.75	0.89	0.94	0.78	0.84
THR	367	0.67	0.66	1.27	1.17	0.57	0.87
VAL	238	1.65	1.69	1.39	1.32	0.48	0.42
TRP	177	3.32	2.79	2.39	3.24	1.17	1.57
TYR	168	2.32	2.12	2.56	2.39	1.41	1.69
**Overall**	5199	1.64	1.65	2.19	2.09	1.25	1.31

Table S2. Comparison between Mean Absolute Errors of χ angles for MCMC (rotamer), MCMC (off-rotamer), GA (rotamer initialization), GA (random initialization), Rosetta Packer and FoldX. The values were obtained by averaging MAE values for 100 high-resolution (≤1.5 Å) PDB structures.

	MAE, ◦																	
	MCMC (rotamer)			MCMC (off-rotamer)			GA (rotamer)			GA (random)			Rosetta packer			FoldX		
AA	χ_1	χ_2	χ_3	χ_1	χ_2	χ_3	χ_1	χ_2	χ_3	χ_1	χ_2	χ_3	χ_1	χ_2	χ_3	χ_1	χ_2	χ_3
ARG	25.2	29.7	67.1	27.4	31.1	65.4	30.1	34.7	63.1	30.2	34.7	59.1	26.2	26.9	56.1	28.4	30.1	62.7
LYS	32.4	31.8	41.4	33.2	38.8	42.4	31.1	38.8	43.4	29.9	38.8	42.4	25.6	31.1	38.2	29.5	31.6	39.2
PHE	15.0	17.4		14.8	19.1		13.9	18.4		15.8	19.7		12.9	13.1		14.1	14.7	
TYR	13.2	13.7		13.0	12.7		14.1	13.2		15.0	12.2		10.7	10.9		13.9	12.4	
TRP	17.6	36.3		18.6	40.3		21.6	42.5		16.3	34.3		12.8	24.5		15.1	32.7	
HIS	22.1	71.8		21.5	70.4		25.1	72.3		26.1	78.8		17.7	66.3		21.7	69.5	
GLU	39.4	41.7		40.8	41.7		42.2	42.6		44.0	43.0		33.1	38.5		35.7	39.3	
GLN	27.3	39.2	52.2	26.4	42.3	51.5	29.7	40.1	54.6	31.3	43.2	53.5	23.4	34.2	48.7	27.9	42.1	51.6
MET	19.8	30.4	46.7	20.1	32.1	47.3	23.5	37.6	58.3	22.2	35.3	62.1	20.0	24.1	49.5	28.3	44.2	55.3
ASN	23.0	48.2		22.7	46.2		31.9	54.4		30.6	52.1		20.1	41.9		22.5	45.0	
ASP	19.2	21.4		22.9	23.4		24.5	26.3		27.8	29.4		21.8	20.2		23.4	23.6	
SER	40.5			42.0			45.8			47.1			37.2			39.0		
LEU	13.0	16.1		15.9	18.0		21.3	22.7		23.9	24.6		11.6	19.0		14.1	17.7	
THR	15.8			16.0			19.8			18.5			15.3			16.8		
ILE	24.6	29.8		27.3	30.5		29.1	32.0		28.7	29.8		9.5	20.8		11.1	22.5	
CYS	23.4			24.0			27.2			29.4			14.5			19.8		
VAL	30.1			32.3			27.1			26.3			13.1			16.0		
PRO	9.6			8.5			19.7			19.0			8.1			10.3		
SEP	19.2	40.4	49.3	27.5	47.6	58.2	25.3	48.9	53.1	23.1	50.9	60.1	20.1	41.9	53.1	27.5	45.0	54.4
TPO	27.9	40.5	61.2	30.2	53.2	65.0	33.0	57.9	64.2	33.0	57.9	64.2	25.1	40.6	55.9	28.2	49.0	59.0
PTR	39.2	64.0	65.5	49.2	67.1	68.8	52.9	70.1	69.5	40.3	62.1	65.5	27.2	50.6	61.9	-	-	-
MLY	26.1	42.5	52.6	29.9	50.1	52.6	27.4	55.3	60.6	24.1	40.5	51.0	32.1	54.2	55.3	-	-	-
CSO	20.2	25.4		24.3	27.5		28.3	32.5		30.9	33.7		27.6	29.3		-	-	-

Table S3. Validation set

PDB ID	Resolution, Å	PTM	Chain	Site
7axn	1.4	SEP	P	72
5o0e	1.5	SEP	A	139
5n3q	1.31	SEP	A	139
6yps	1.35	SEP	A	139
7d0e	1.4	SEP	B	104
6yia	1.3	SEP	P	127
7nzk	1.4	SEP	P	45
6esa	1.31	SEP	A	338
7njb	1.4	SEP	P	45
7bkh	1.4	SEP	P	45
5n3h	1.36	SEP	A	139
5my9	1.33	SEP	P	935
7bjl	1.4	SEP	P	45
5lvl	1.4	SEP	A	241
7np2	1.27	SEP	P	175
6y18	1.3	SEP	B	179
6qhm	1.25	SEP	P	281

7nxw	1.2	SEP	P	45
7ndy	1.44	SEP	A	4
5n3t	1.21	SEP	A	139
4u1p	1.4	PTR	B	4
7ll4	1.31	PTR	A	1007
1sha	1.5	PTR	B	201
5gjh	1.2	PTR	B	191
7rnu	1.45	PTR	B	53
4yll	1.4	PTR	A	321
7cio	1.1	PTR	B	191
3u3z	1.5	PTR	B	4
2src	1.5	PTR	A	527
1fmk	1.5	PTR	A	527
1lkk	1	PTR	B	252
3wa4	1.35	PTR	B	191
4yjr	1.32	PTR	A	525
4yjq	1.34	PTR	A	525
6s1b	1.3	PTR	A	321
6a1f	1.5	PTR	A	321
2vif	1.45	PTR	P	568
5gji	0.9	PTR	B	191
3bux	1.35	PTR	A	1003
4iva	1.5	PTR	A	1008
6sut	1.2	TPO	A	285
7b9r	1.15	TPO	B	594
6yqi	1.42	TPO	A	197
6rwu	1.46	TPO	P	387
4gv1	1.49	TPO	A	308
5ylf	1.5	TPO	A	70
5lcp	1.43	TPO	A	197
3a7j	1.5	TPO	A	178
5b1a	1.5	TPO	T	11
5n3k	1.33	TPO	A	197
5n33	1.43	TPO	A	197
2q5a	1.5	TPO	B	502
5ylc	1.5	TPO	A	70
5mhi	1.49	TPO	A	197
7ba9	1.48	TPO	B	594
6spu	1.39	TPO	A	197
3a7i	1.45	TPO	A	178
7ba6	1.4	TPO	B	594
5n3r	1.36	TPO	A	197
6y3m	1.5	TPO	P	391
4yav	1.4	CSO	A	94
7bkh	1.4	CSO	A	38

6xaj	1.5	CSO	A	178
7nk3	1.4	CSO	A	38
3non	1.05	CSO	A	101
5ufh	1.45	CSO	A	87
2vr8	1.36	CSO	F	111
7biw	1.2	CSO	A	38
7nla	1.4	CSO	A	38
3f71	1.2	CSO	A	106
4d6a	1.45	CSO	A	117
2zpb	1.3	CSO	A	114
5aui	1.5	CSO	A	86
6qtv	1.31	CSO	A	510
4p36	1.18	CSO	A	106
3iix	1.25	CSO	A	183
6qtq	1.3	CSO	A	492
7nmh	1.4	CSO	A	38
6ni9	1.2	CSO	B	101
1k3i	1.4	CSO	A	228
6f7w	1.28	MLY	A	83
5em1	1.45	MLY	A	88
4wri	1.4	MLY	A	29
5m2f	1.5	MLY	X	263
3lgf	1.5	MLY	B	370
6stz	1.14	MLY	D	79
2zpm	0.98	MLY	A	237
4fr9	1.2	MLY	A	137
5jh8	1.02	MLY	A	233
3mc3	1.49	MLY	A	89
3ln3	1.18	MLY	A	33
3f9x	1.25	MLY	G	20
4gnr	1	MLY	A	345
4up0	1.28	MLY	F	4
7kqw	0.93	MLY	A	163
6bhd	1.25	MLY	B	9
4ee6	1.33	MLY	B	135
6xzl	1.39	MLY	A	329
5tdr	1.42	MLY	B	4
4wev	1.45	MLY	X	33
